# Supplementary material for: Wnt/β-catenin signalling is required for pole-specific chromatin remodeling during planarian regeneration
Source: Nat Commun. 2023 Jan 18;14:298. doi: 10.1038/s41467-023-35937-y (PMC9849279; doi:10.1038/s41467-023-35937-y)
Supplement: Supplementary file 1 — Supplementary Information [file 41467_2023_35937_MOESM1_ESM.pdf]

Wnt/β-catenin signalling is required for pole-specific chromatin remodeling during planarian regeneration

Eudald Pascual-Carreras[1], Marta Marín-Barba[2], Sergio Castillo-Lara[1], Pablo Coronel-Córdoba[1], Marta Silvia Magri[3], Grant N. Wheeler[2], Jose Luis Gomez-Skarmeta[3], Josep F. Abril[1], Emili Saló[1*] & Teresa Adell[1*]

[1] Department of Genetics, Microbiology and Statistics, Universitat de Barcelona (UB) & Institute of Biomedicine of Universitat de Barcelona (IBUB), Barcelona, Spain.

[2] School of Biological Sciences, University of East Anglia, Norwich Research Park, Norwich, UK.

[3] Centro Andaluz de Biología del Desarollo (CABD), Universidad Pablo de Olavide, Sevilla, Spain.

[*]Corresponding authors: Emili Saló (esalo@ub.edu) and Teresa Adell (tadellc@ub.edu)

Supplementary information

Supplementary Data 1-10

Supplementary Figure 1-10

Supplementary Data 1.

Specific accessible chromatin regions (ACR) of anterior and posterior wounds at 12 hR contain different TFs binding sites. For each ACR, it is shown the scaffold location, its position within, the fold change with respect to the data from the opposite wound and the FDR statistic value. We also show the specific putative enhancers of anterior and posterior wounds at 12 hR. For each specific putative anterior and posterior enhancer, it is shown the scaffold location, its position within and how it behaves in *notum* or *wnt1* (RNAi) conditions; being: accessible, slightly accessible, less accessible or non-accessible.

Supplementary Data 2.

The transcription factor binding motif presence in the anterior and posterior facing wounds at 12 hours of regeneration. For each wound site, TF presence was explored in the putative enhancer and promoter regions. The consensus sequence, *p*-value (generated by HOMER), log *p*-value, *q*-value (Benjamini), number of target sequences with the motif, percentage of sequences with the motif, number of background sequences with the motif and percentage of background sequences with the motif is shown per TF.

Supplementary Data 3.

Results of the *wnt1* (RNAi) RNA-seq experiment. 'Dif Expressed' shows all genes up- and downregulated in the different regenerative time points. 'Downregulated' shows the genes downregulated in the different regenerative time points. It includes the presence of TCF motifs in the CREs (promoters and enhancers) and their presence in the dataset[1]. LogFC, adjusted *p*-value (FDR on lima-voom empirical Bayes moderated t-test), genome ID[2], condition (regenerative time point), gene name, Transcriptome ID[3] and human homolog is shown per each gene.

Supplementary Data 4.

Transcription factor motifs presence in the putative promoters and enhancers of the *wnt1* downregulated genes. Motif name, sequence consensus, *p*-value (generated by HOMER), log *p*-value, q-value (Benjamini), number of target sequences with the motif, percentage of target sequences with the motif, number of background sequences with the motif and percentage of background sequences with the motif are shown.

Supplementary Data 5.

wnt1 (RNAi) RNA-seq downregulated genes with a TCF binding site in its promoter or enhancer region. Gene ontology enrichment analysis of the genes downregulated in *wnt1* (RNAi) animals and containing TCF binding sites is shown, list of the enriched categories showing the significance of the enrichment is shown. The R package topGO with Fisher's exact test and adjusted *p*-value <0.05 (FDR) was used for the analysis.

Supplementary Data 6.

Transcription factor binding sites found in Enhancer 1 (E1) and Enhancer 2 (E2). The DNA sequence, the motif name, the strand and the motif score were displayed for each. SLP1 (FOXG) motif was highlighted in yellow.

Supplementary Data 7.

Presence of accessible chromatin regions (ACRs) with ATAC-seq or DNase-seq evidence, active enhancers with H3K27ac and H3K4me1/2/3 evidence. – indicated no evidence. The genome source per each species was added. The presence of FOXG binding sites in the first intron of *wnt1* gene in different species was determined using the FIMO tool. N indicates no evidence, Y indicates evidence, and the number of motifs found is also indicated, depending on the *Homo sapiens* (*Hsap*) or *Drosophila melanogaster* (*Dmel*) FOXG matrix used.

Supplementary Data 8.

FoxG binding site motif presence in the CREs of the wound-induced genes[4], indicating its localization: Proximal C.R.E or Enhancer.

Supplementary Data 9.

List of genes with CREs specifically accessible in posterior and anterior wounds (12 hours of regeneration), containing a FOXG binding site motif. Gene ontology (GO) list of the mentioned genes. GOs related to Biological Process (BP) and Molecular Function (ML) were shown per condition (related to Supplementary Fig. 10). Per each GO, the GO term and genome ID were indicated. Per each gene linked to a GO, the logFC value, adjusted *p*-value (FDR over topGO Fisher's exact test), and z-score indicating more presence of FOXG binding sites in the putative enhancer or in the promoter region per gene were indicated.

Supplementary Data 10.

List of primers used in this study. We display the name of the primer, the technique where they were used and the sequences forward (Fw) and reverse (Rv) 5’ to 3’.

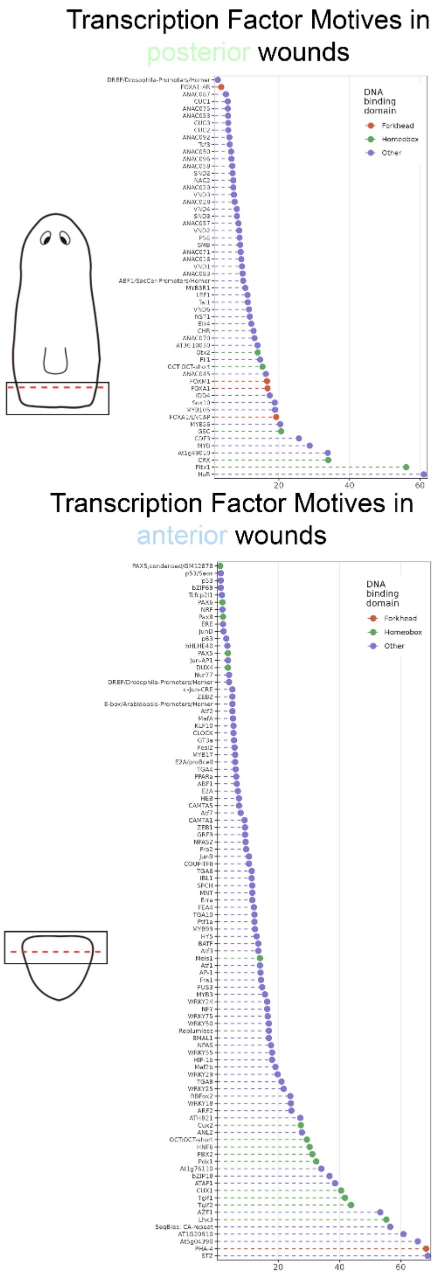

Supplementary Figure 1. The transcription factor (TF) binding motif presence in the anterior (bottom) and posterior (top) facing wounds at 12 hR. For each wound site, TF binding motif presence was explored in the putative enhancer and promoter regions. The X axis shows the percentage of genes with the TF binding motif presence in their CREs. Per condition, TF are ordered by percentage of sequences with significant motifs in the putative enhancers. Different colors and shapes indicate TF families.

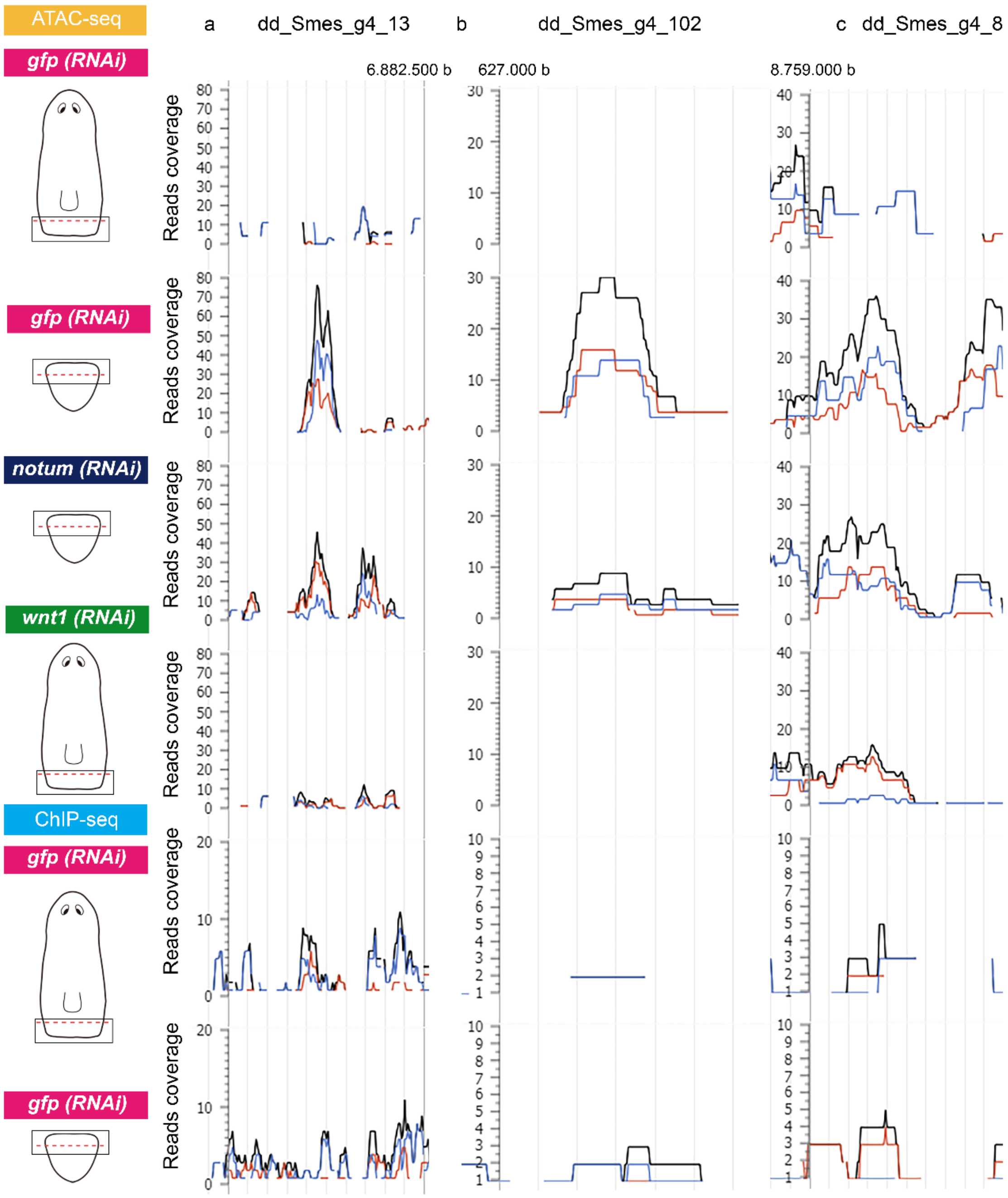

Supplementary Figure 2. Anterior putative active enhancers show different chromatin accessibility in *notum* and *wnt1* (RNAi) wound regions. **a** The anterior enhancer presents less accessibility in *notum* (RNAi) wound cells, while in *wnt1* (RNAi) shows none. **b** The specific anterior enhancer, in the anterior *notum* (RNAi) wound cells has become non-accessible, and the same occurs in the posterior *wnt1* (RNAi) wound cells. **c** The chromatin is less accessible in the anterior *notum* (RNAi) wound cells, being accessible in the posterior *wnt1* (RNAi) wound cells. For each example, the genome scaffold is indicated together with the closest base reference. The read coverage scale has been adjusted for each example. Replicates are shown in blue and red lines; black lines

represent the added projection. ATAC-seq and ChIP-seq tracks were retrieved from genome browser at https://compgen.bio.ub.edu/jbrowse/.

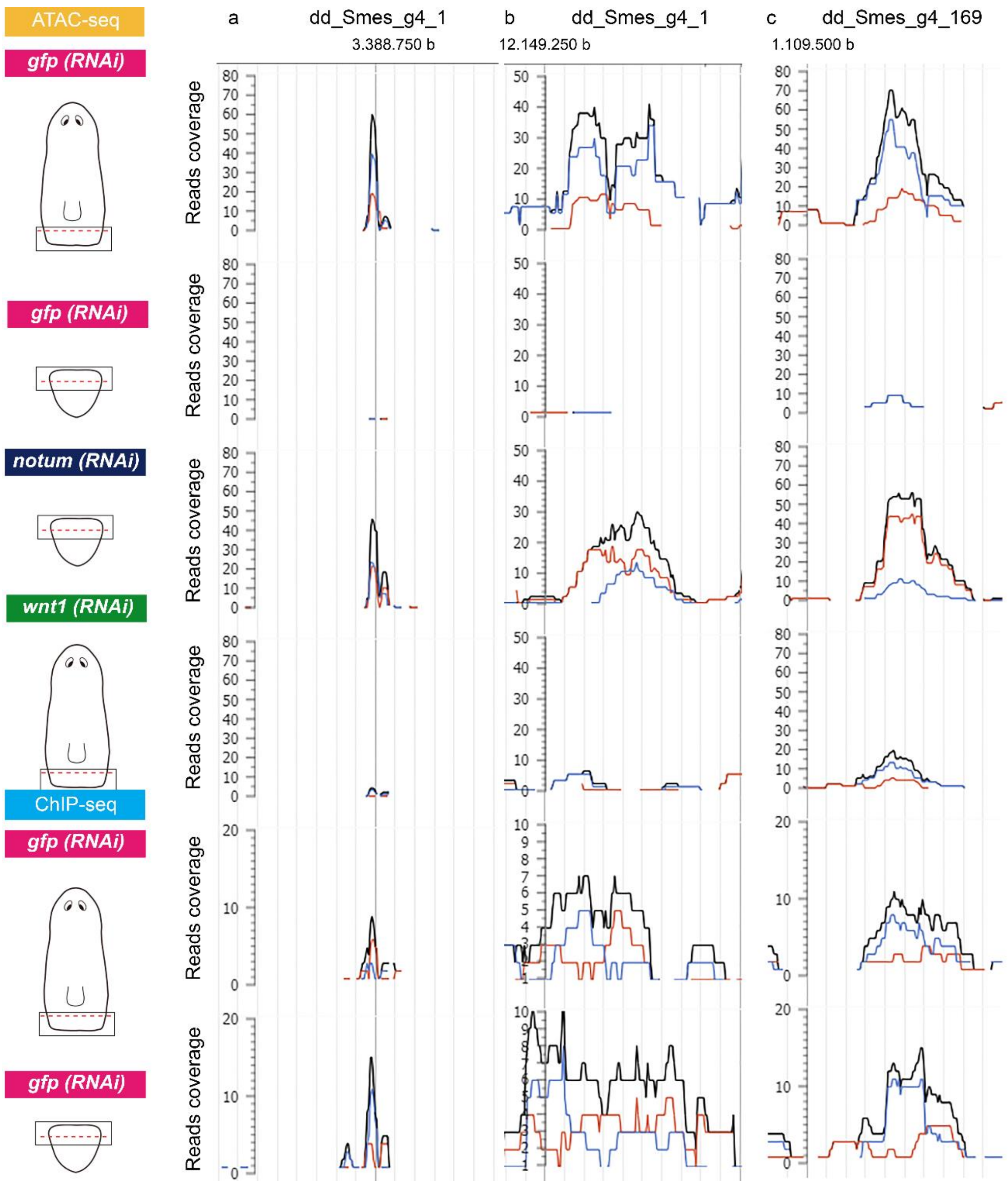

Supplementary Figure 3. Posterior putative active enhancers show different chromatin accessibility in *notum* and *wnt1* (RNAi) wound regions. **a** The posterior enhancer is accessible in *notum* (RNAi) wound cells, while in *wnt1* (RNAi) is not. **b** The posterior enhancer in *notum* (RNAi) wound cells present a slight increment of the chromatin accessibility; being non-accessible in *wnt1* (RNAi) wound cells. **c** The chromatin accessibility of the posterior enhancer has slightly increased in *notum* (RNAi), and has maintained accessibility in *wnt1* (RNAi) wound cells, respectively. For each example, the genome scaffold is indicated together with the closest base reference. The read coverage scale has been adjusted for each example. Replicates are shown in blue and

red lines; black lines represent the added projection. ATAC-seq and ChIP-seq tracks were retrieved from genome browser at https://compgen.bio.ub.edu/jbrowse/.

Posterior putative active enhancers

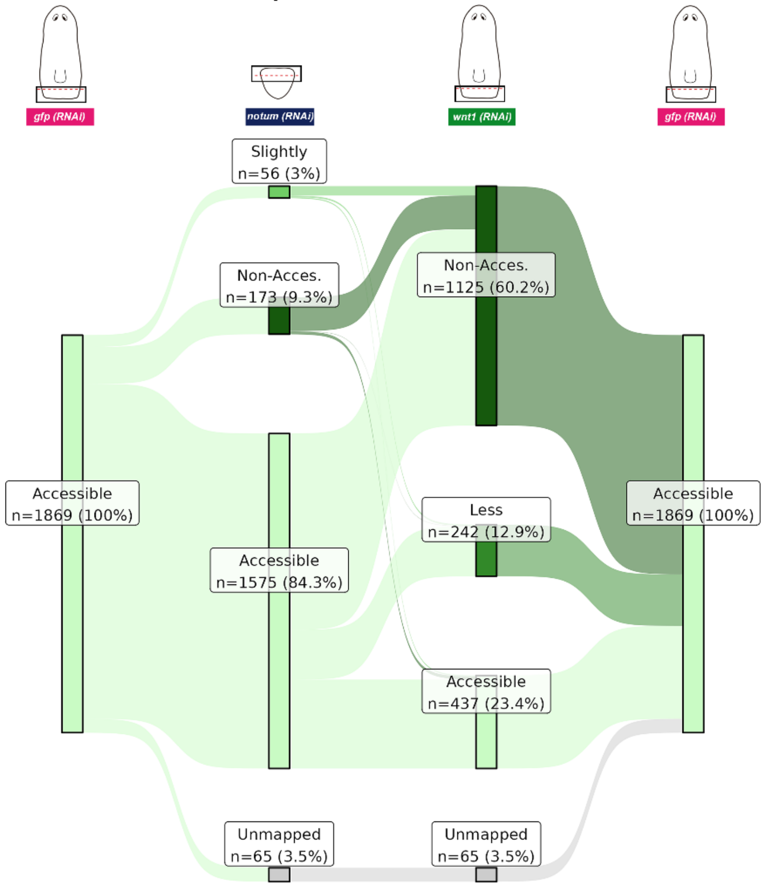

Anterior putative active enhancers

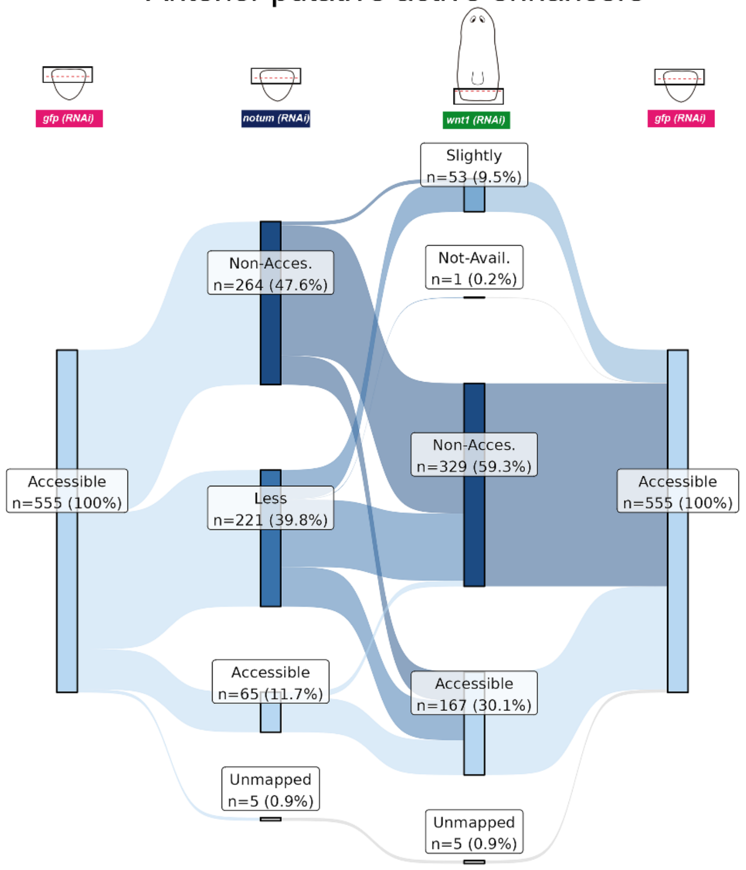

Supplementary Figure 4. Chromatin accessibility of posterior (top) and anterior (bottom) active enhancer regions in *notum* and *wnt1* (RNAi) conditions. Absolut numbers and percentages are shown per experimental condition. All the data for this figure is provided in Supplementary Data 1.

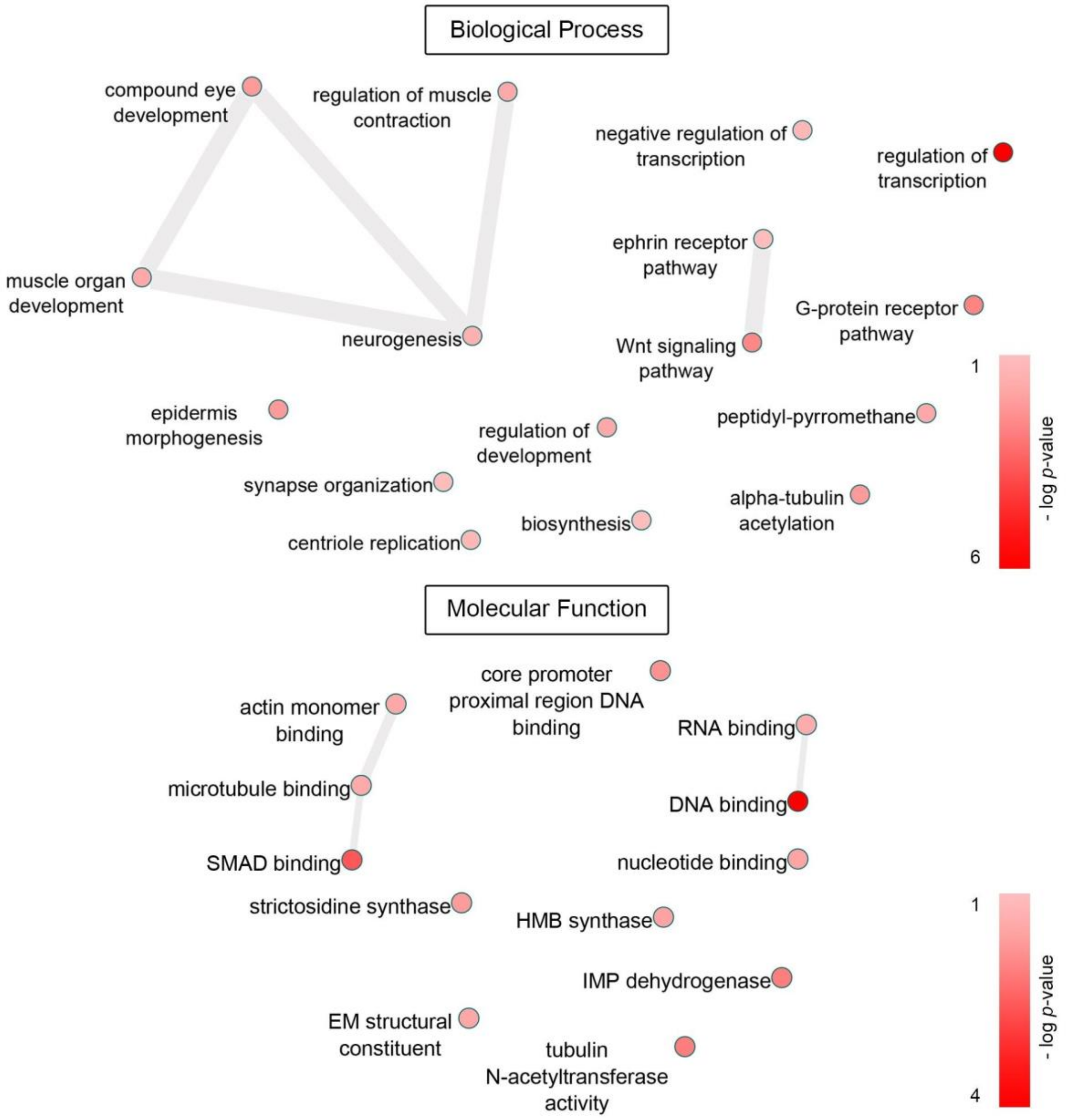

Supplementary Figure 5. Gene ontology enrichment analysis of the genes downregulated in *wnt1* (RNAi) animals and containing TCF binding sites visualized by ReviGO[5] (http://revigo.irb.hr/, default terms) and Cytoscape[6]. Gene ontology was analyzed according to Biological Process (top) and Molecular Function (bottom). Circle color indicates the *P*-value of each term (according to topGO Fisher's exact test). Highly similar GO terms are linked by edges in the graph.

>dd_Smes_g4_197 dd_Smes_g4_197:263306..277425 (+ strand) class=gene length=14120

[illegible]

CDS wnt1
Enhancer wnt1
foxG motif

Supplementary Figure 6. Genomic sequence of *Smed-wnt1* locus showing the genomic organization of the gene (exons in green), the enhancers in the first intron (in yellow) and the FoxG binding motifs (grey).

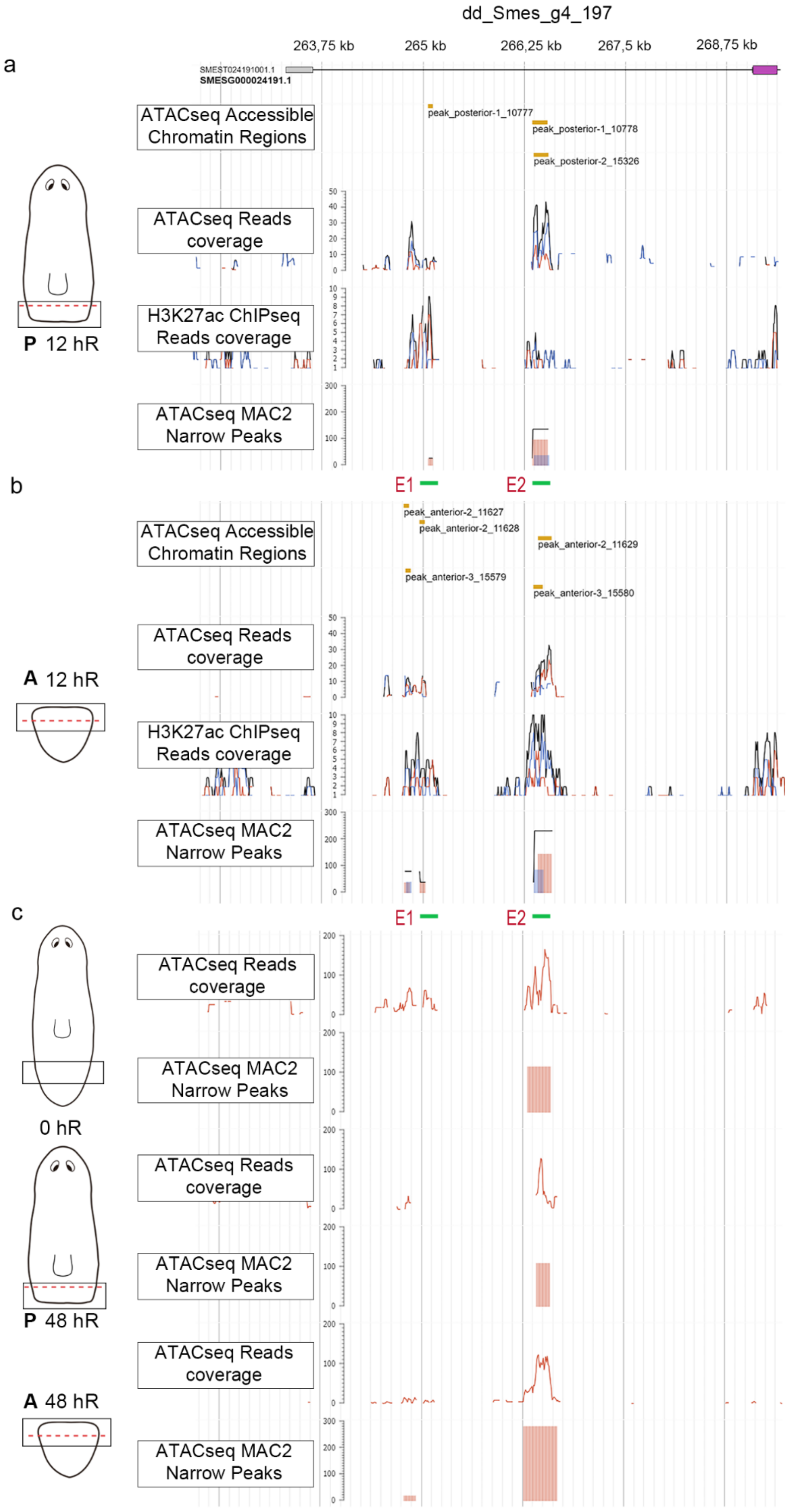

dd_Smes_g4_197
263,75 kb
265 kb
266,25 kb
267,5 kb
268,75 kb
a
SMEST024191001.1
SMESG000024191.1
ATACseq Accessible Chromatin Regions
peak_posterior-1_10777
peak_posterior-1_10778
peak_posterior-2_15326
ATACseq Reads coverage
H3K27ac ChIPseq Reads coverage
ATACseq MAC2 Narrow Peaks
P 12 hR
E1
E2
b
ATACseq Accessible Chromatin Regions
peak_anterior-2_11627
peak_anterior-2_11628
peak_anterior-2_11629
peak_anterior-3_15579
peak_anterior-3_15580
ATACseq Reads coverage
A 12 hR
H3K27ac ChIPseq Reads coverage
ATACseq MAC2 Narrow Peaks
E1
E2
c
ATACseq Reads coverage
ATACseq MAC2 Narrow Peaks
0 hR
ATACseq Reads coverage
ATACseq MAC2 Narrow Peaks
P 48 hR
ATACseq Reads coverage
A 48 hR
ATACseq MAC2 Narrow Peaks

Supplementary Figure 7. *Wnt1* first intron enhancer accessibility in posterior and anterior wounds. A Genome Browser screenshot showing ATAC-seq Accessible Chromatin Regions (ACR), ATAC-seq reads coverage, H3K27ac ChIP read coverage and ATAC-seq MAC2 narrow peaks of the posterior wounds at 12 hR. **b** Genome Browser screenshot showing ATAC-seq Accessible Chromatin Regions (ACR), ATAC-seq reads coverage, H3K27ac ChIP read coverage and ATAC-seq MAC2 narrow peaks of the anterior wounds at 12 hR. Replicates are shown in blue and red lines, black lines represent the added projection. **C** Genome Browser screenshot showing the ATAC-seq reads coverage and ATAC-seq MAC2 narrow peaks of unwounded animals (0 hR) and the posterior and anterior wounds at 48 hR. One replicate was generated per sample. For each example, the genome scaffold is indicated together with the closest base reference. The read coverage scale has been adjusted for each example. ATAC-seq and ChIP-seq tracks were retrieved from genome browser at https://compgen.bio.ub.edu/jbrowse/.

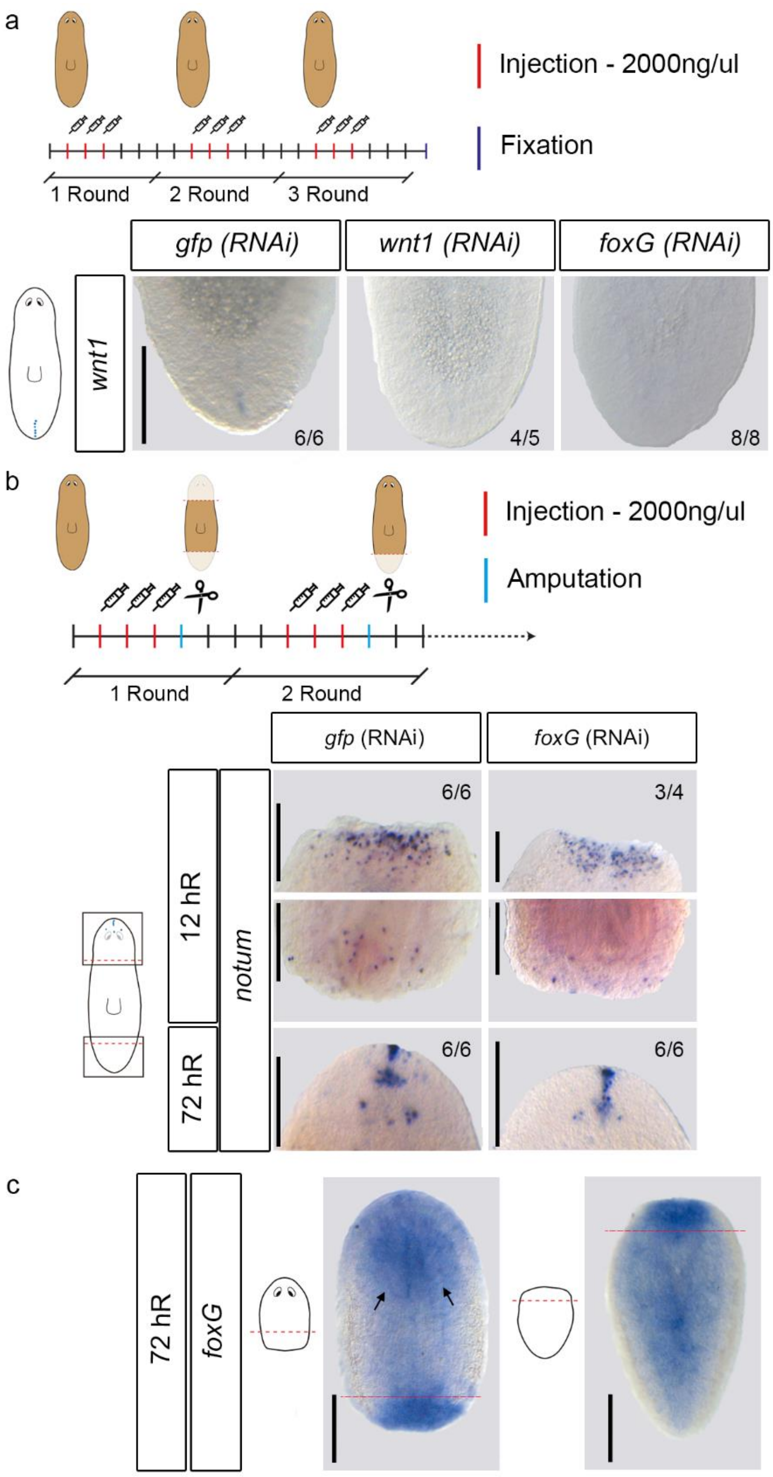

Supplementary Figure 8. *foxG* controls *wnt1* but not *notum*, expression. A WISH of *wnt1* in intact animals after *wnt1* and *foxG* inhibition reveal its absence. Data representative of one experiment. A scheme of the RNAi experimental design is shown. **B** WISH of *notum* in regenerating *foxG* (RNAi) showed no differences between *gfp* and *foxG* (RNAi) animals. Data representative of one experiment. A scheme of the RNAi experimental design is shown. **C** WISH of *foxG* in regenerating anterior and posterior

wounds at 72 hR. Data representative of one experiment. Red dashed line indicates the amputation plane. Black arrows point to the lobbed cephalic ganglia. Scale bar: 500 µm.

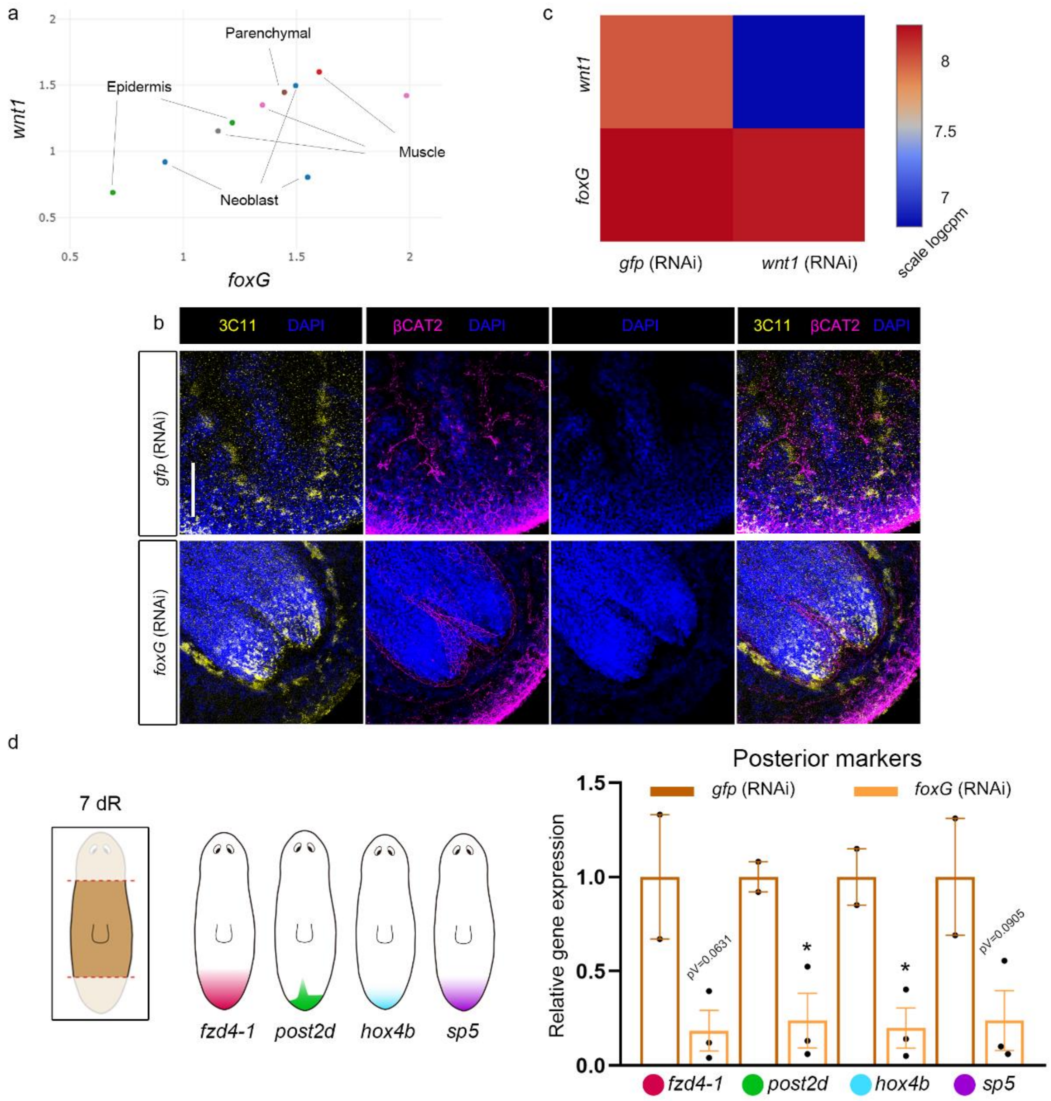

Supplementary Figure 9. *foxG* (RNAi) animals showed lack of posterior regeneration. A SC-seq data from[7] demonstrates co-expression of *wnt1* and *foxG* mainly in muscular and neuronal cells. **B** Tailless phenotype in *foxG* (RNAi) animals. **C** Heat map expression of *foxG* and *wnt1* in *wnt1* (RNAi) RNA-seq reveals that *foxG* is not downregulated. **D** qRT-PCR analysis quantifying posterior markers in *foxG* (RNAi) animalsat 7 days of regeneration (dR), showing their expression reduction. Mean relative expression is plotted as $2^{-\Delta\Delta CT}$ values, and error bars are SEM. Student's *t* test (unpaired, two-tailed) **P*<0.0301 (*post2d*) and **P*<0.02 (*hox4b*). Biological replicates: controls, n=2; RNAi, n=3, in one experiment. Values are provided in Source Data file. Schematic illustrations of posterior markers were added. Scale bar: 100 µm.

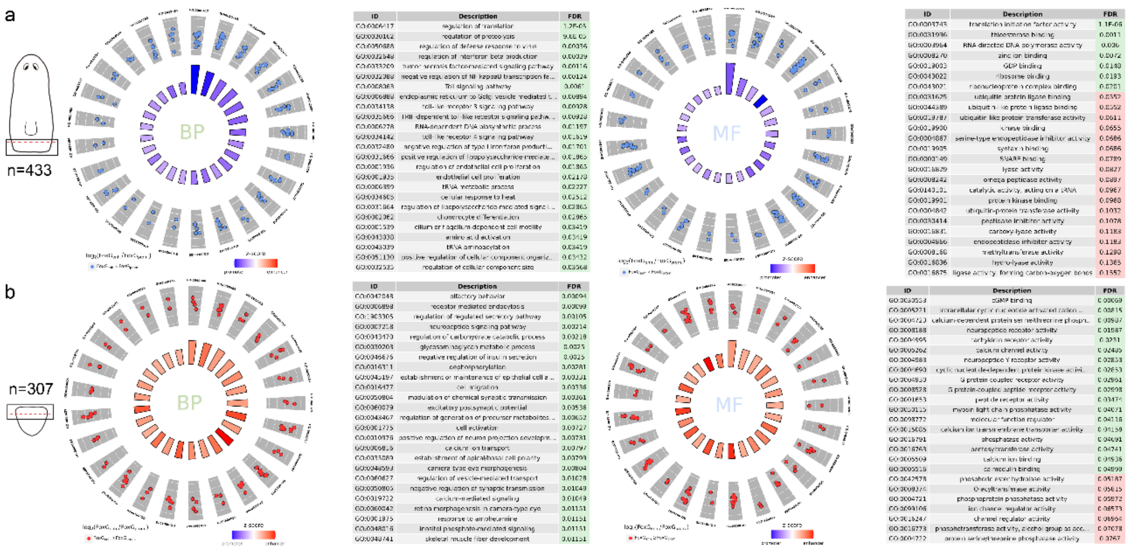

Supplementary Figure 10. Gene ontology of genes presenting a FOXG binding site in their *cis*-regulatory elements. A Gene ontology (GO) of genes with FOXG binding site presence in specific accessible CREs in posterior wounds. **B** Gene ontology (GO) of genes with FOXG binding site presence in specific accessible CREs in anterior wounds. Number of genes with FOXG binding sites in their CREs are shown per condition. Top 25 Gos related to Biological Process (BP) and Molecular Function (ML) were shown per condition (expanded in supplementary Data 9). In each GO plot, logFC value indicating a higher presence of FOXG binding sites in the putative enhancer or in the promoter region per gene was shown in the external ring. Each dot represents a gene, red and blue dots present more FOXG binding sites in the putative enhancer region or in the promoter region, respectively. The logFC value indicating a higher presence of FOXG binding sites in the putative enhancer or in the promoter region presents an associated adjusted *p*-value (FDR over topGO Fisher's exact test) and a z-score, represented by bar heights and colour in the inner ring, respectively.

References

1. Tewari, A. G., Owen, J. H., Petersen, C. P., Wagner, D. E. & Reddien, P. W. A small set of conserved genes, including sp5 and Hox, are activated by Wnt signaling in the posterior of planarians and acoels. *PLOS Genet.* **15**, e1008401 (2019).

2. Grohme, M. A. *et al.* The genome of Schmidtea mediterranea and the evolution of core cellular mechanisms. *Nature* **554**, 56–61 (2018).

3. Rozanski, A. *et al.* PlanMine 3.0-improvements to a mineable resource of flatworm biology and biodiversity. *Nucleic Acids Res.* **47**, (2019).

4. Wurtzel, O. *et al.* A Generic and Cell-Type-Specific Wound Response Precedes Regeneration in Planarians. *Dev. Cell* **35**, 632–645 (2015).

5. Supek, F., Bošnjak, M., Škunca, N. & Šmuc, T. Revigo summarizes and visualizes long lists of gene ontology terms. *PLoS One* **6**, e21800 (2011).

6. Shannon, P. *et al.* Cytoscape: A Software Environment for Integrated Models of Biomolecular Interaction Networks. *Genome Res.* **13**, 2498–2504 (2003).

7. Fincher, C. T., Wurtzel, O., de Hoog, T., Kravarik, K. M. & Reddien, P. W. Cell type transcriptome atlas for the planarian *Schmidtea mediterranea*. *Science (80-.).* **5**, eaaq1736 (2018).
