# Supplementary material for: Wnt/β-catenin signalling is required for pole-specific chromatin remodeling during planarian regeneration
Source: Nat Commun. 2023 Jan 18;14:298. doi: 10.1038/s41467-023-35937-y (PMC9849279; doi:10.1038/s41467-023-35937-y)
Supplement: Supplementary file 3 — Description of Additional Supplementary Files [file 41467_2023_35937_MOESM3_ESM.pdf]

Description of Additional Supplementary Files

File Name: Supplementary Data 1
Description: Specific accessible chromatin regions (ACR) of anterior and posterior wounds at 12 hR contain different TFs binding sites. For each ACR, it is shown the scaffold location, its position within, the fold change with respect to the data from the opposite wound and the FDR statistic value. We also show the specific putative enhancers of anterior and posterior wounds at 12 hR. For each specific putative anterior and posterior enhancer, it is shown the scaffold location, its position within and how it behaves in *notum* or *wnt1* (RNAi) conditions; being: accessible, slightly accessible, less accessible or non-accessible.

File Name: Supplementary Data 2
Description: The transcription factor binding motif presence in the anterior and posterior facing wounds at 12 hours of regeneration. For each wound site, TF presence was explored in the putative enhancer and promoter regions. The consensus sequence, *p*-value (generated by HOMER), log *p*-value, *q*-value (Benjamini), number of target sequences with the motif, percentage of sequences with the motif, number of background sequences with the motif and percentage of background sequences with the motif is shown per TF.

File Name: Supplementary Data 3
Description: Results of the *wnt1* (RNAi) RNA-seq experiment. 'Dif Expressed' shows all genes up-and downregulated in the different regenerative time points. 'Downregulated' shows the genes downregulated in the different regenerative time points. It includes the presence of TCF motifs in the CREs (promoters and enhancers) and their presence in the dataset[1]. LogFC, adjusted *p*-value (FDR on lima-voom empirical Bayes moderated t-test), genome ID[2], condition (regenerative time point), gene name, Transcriptome ID[3] and human homolog is shown per each gene.

File Name: Supplementary Data 4
Description: Transcription factor motifs presence in the putative promoters and enhancers of the *wnt1* downregulated genes. Motif name, sequence consensus, *p*-value (generated by HOMER), log *p*-value, q-value (Benjamini), number of target sequences with the motif, percentage of target sequences with the motif, number of background sequences with the motif and percentage of background sequences with the motif are shown.

File Name: Supplementary Data 5
Description: *wnt1* (RNAi) RNA-seq downregulated genes with a TCF binding site in its promoter or enhancer region. Gene ontology enrichment analysis of the genes downregulated in *wnt1* (RNAi) animals and containing TCF binding sites is shown, list of the enriched categories showing the significance of the enrichment is shown. The R package topGO with Fisher's exact test and adjusted *p*-value <0.05 (FDR) was used for the analysis.

File Name: Supplementary Data 6
Description: Transcription factor binding sites found in Enhancer 1 (E1) and Enhancer 2 (E2). The DNA sequence, the motif name, the strand and the motif score were displayed for each. SLP1 (FOXG) motif was highlighted in yellow.

File Name: Supplementary Data 7
Description: Presence of accessible chromatin regions (ACRs) with ATAC-seq or DNase-seq evidence, active enhancers with H3K27ac and H3K4me1/2/3 evidence. – indicated no evidence. The genome source per each species was added. The presence of FOXG binding sites in the first intron of *wnt1* gene in different species was determined using the FIMO tool. N indicates no evidence, Y indicates evidence, and the number of motifs found is also indicated, depending on the *Homo sapiens* (*Hsap*) or *Drosophila melanogaster* (*Dmel*) FOXG matrix used.

File Name: Supplementary Data 8
Description: FoxG binding site motif presence in the CREs of the wound-induced genes[4], indicating its localization: Proximal C.R.E or Enhancer.

File Name: Supplementary Data 9
Description: List of genes with CREs specifically accessible in posterior and anterior wounds (12 hours of regeneration), containing a FOXG binding site motif. Gene ontology (GO) list of the mentioned genes. GOs related to Biological Process (BP) and Molecular Function (ML) were shown per condition (related to Supplementary Fig. 10). Per each GO, the GO term and genome ID were indicated. Per each gene linked to a GO, the logFC value, adjusted *p*-value (FDR over topGO Fisher's exact test), and z-score indicating more presence of FOXG binding sites in the putative enhancer or in the promoter region per gene were indicated.

File Name: Supplementary Data 10
Description: List of primers used in this study. We display the name of the primer, the technique where they were used and the sequences forward (Fw) and reverse (Rv) 5’ to 3’.
