# Supplementary material for: Wnt/β-catenin signalling is required for pole-specific chromatin remodeling during planarian regeneration
Source: Nat Commun. 2023 Jan 18;14:298. doi: 10.1038/s41467-023-35937-y (PMC9849279; doi:10.1038/s41467-023-35937-y)
Supplement: Supplementary file 14 — Reporting Summary [file 41467_2023_35937_MOESM14_ESM.pdf]

nature portfolio

Corresponding author(s): Teresa Adell

Last updated by author(s): Dec 21, 2022

Reporting Summary

Nature Portfolio wishes to improve the reproducibility of the work that we publish. This form provides structure for consistency and transparency in reporting. For further information on Nature Portfolio policies, see our Editorial Policies and the Editorial Policy Checklist.

Statistics

For all statistical analyses, confirm that the following items are present in the figure legend, table legend, main text, or Methods section.

n/a	Confirmed	
☐	☒	The exact sample size (*n*) for each experimental group/condition, given as a discrete number and unit of measurement
☐	☒	A statement on whether measurements were taken from distinct samples or whether the same sample was measured repeatedly
☐	☒	The statistical test(s) used AND whether they are one- or two-sided<br>*Only common tests should be described solely by name; describe more complex techniques in the Methods section.*
☒	☐	A description of all covariates tested
☐	☒	A description of any assumptions or corrections, such as tests of normality and adjustment for multiple comparisons
☐	☒	A full description of the statistical parameters including central tendency (e.g. means) or other basic estimates (e.g. regression coefficient) AND variation (e.g. standard deviation) or associated estimates of uncertainty (e.g. confidence intervals)
☐	☒	For null hypothesis testing, the test statistic (e.g. *F*, *t*, *r*) with confidence intervals, effect sizes, degrees of freedom and *P* value noted<br>*Give P values as exact values whenever suitable.*
☒	☐	For Bayesian analysis, information on the choice of priors and Markov chain Monte Carlo settings
☒	☐	For hierarchical and complex designs, identification of the appropriate level for tests and full reporting of outcomes
☒	☐	Estimates of effect sizes (e.g. Cohen's *d*, Pearson's *r*), indicating how they were calculated

Our web collection on statistics for biologists contains articles on many of the points above.

Software and code

Policy information about availability of computer code

Data collection	FoxG binding site presence: USCS browser. Image acquisition: in vivo images were acquired with X. Brightfield colorimetric ISH images obtained with a ProgRes C3 camera from Jenoptik (Jena, TH, Germany). A Zeiss LSM 880 confocal microscope (Zeiss, Oberkochen, Germany) was used to obtain confocal images of whole-mount immunostainings.
Data analysis	ll software versions are listed at the end of this section.<br><br>ATAC-seq and ChIPmentation: Reads were aligned by bowtie. Reads were filtered from BAM files. BAM were converted to BED; for the ATAC-seq replicates then the coordinates on the BED files were shifted +4 and −5 positions to overcome the Tn5 cut position. MACS2 was used for peak calling and HOMER for motif discovery. Differential binding analysis was carried out using DiffBind R functions. ATAC-seq comparison ACR between anterior and posterior: ATAC-seq anterior versus posterior comparison were crossmatched by DiffBind. Cis-regulatory elements annotation: both ChIP-seq and ATAC-seq data from all collected samples were used. Narrowpeaks over the genome were identified using MACS2. These peaks were merged using the mergePeaks command of the HOMER software suite. Motif finding: enhancer and promoter regions using the HOMER's findMotifsGenome command. FoxG binding site presence: UCSC browsers. FIMO tool (https://meme-suite.org/meme/tools/fimo) using the default parameters. FASTA files containing DNA sequences corresponding to the wnt1 first intron from different species.<br><br>RNA-seq: RNA reads were mapped against the planarian genome version S2F242 using the STAR software tool. Differentially expressed genes were identified using the lima-voom pipeline. Gene Ontology of DEG were visualized with ReviGO (http://revigo.irb.hr/) and Cytoscape 3.9.1, or PANTHER (http://www.pantherdb.org/). qRT-PCR: 7500 Fast PCR System (Applied Biosystems). Image analysis: Fiji/ImageJ was used to show representative confocal stacks for each experimental condition.

A PDF describing all the Computational Supplementary Methods is available from the following direct link, also referred from GitHub repository: https://compgen.bio.ub.edu/datasets/2022_NatComm/2022_NatComm_BImethods.pdf
All software tools used for the analyses were open source, see versions listed below (and section 9.6.3 of the Computational Supplementary Methods), or custom code (released under GNU General Public License, GNU-GPL v3), which are available through the following GitHub repository: https://github.com/CompGenLabUB/2022_NatComm_BImethods [DOI:10.5281/zenodo.7455255].

Software versions for the Bioinformatic analyses are listed here:
• gawk : GNU Awk 5.1.0
• trimmomatic : 0.32
• perl : 5.34.0
• fastQC : v0.11.2
• python2 : 2.7.16
• STAR : 2.7.0e
• python3 : 3.9.9
• gffread : v0.12.7
• NCBI-BLAST : 2.12.0+
• CD-Hit : 4.6 (built on Jul 16 2015)
• wget : 1.21.2
• BBDuk : 35.14
• EMBOSS : 6.6.0.0 (infoseq)
• samtools : 1.13 (with htslib 1.13+ds)
• bowtie : 1.3.1
• MACS2 : 2.1.2
• htslib : 1.13+ds (tabix)
• bamtools : 2.5.1
• convert2bed : 2.4.40
• bedtools : v2.30.0
• deepTools : 3.5.1
• bedGraphToBigWig : v.4
• HOMER : 4.8.3
• R version 4.1.2 for most of the analyses [edgeR : 3.36.0; limma : 3.50.1; topGO : 2.46.0]

Software versions for the ChIP-Seq Bioinformatic analyses are listed here:
• MACS2 : 2.1.2
• deepTools : 3.5.1
• bowtie : 1.3.1; samtools : 1.13 (with htslib 1.13+ds); bamtools : 2.5.1
• BBDuk : 35.14
• R version 3.5.2 [DiffBind : 2.10.0]; R version 4.1.2 [ATACseqQC : 1.18.0; ChIPpeakAnno : 3.28.1; idr : 1.2]

Further version details can be found on section 9.6.3 and 9.6.4 of the CSM document.

For manuscripts utilizing custom algorithms or software that are central to the research but not yet described in published literature, software must be made available to editors and reviewers. We strongly encourage code deposition in a community repository (e.g. GitHub). See the Nature Portfolio guidelines for submitting code & software for further information.

Data

Policy information about availability of data

All manuscripts must include a data availability statement. This statement should provide the following information, where applicable:

- Accession codes, unique identifiers, or web links for publicly available datasets
- A description of any restrictions on data availability
- For clinical datasets or third party data, please ensure that the statement adheres to our policy

The raw sequencing data sets generated for this research have been deposited with links to BioProject accession number PRJNA800775 [https://www.ncbi.nlm.nih.gov/bioproject/PRJNA800775] in the NCBI BioProject database. The corresponding NCBI Sequence Read Archive (SRA) accessions for the samples included in that BioProject are: for RNA-Seq samples, SRR17766314 [https://www.ncbi.nlm.nih.gov/sra/SRR17766314], SRR17766312 [https://www.ncbi.nlm.nih.gov/sra/SRR17766312], SRR17766311 [https://www.ncbi.nlm.nih.gov/sra/SRR17766311], SRR17766310 [https://www.ncbi.nlm.nih.gov/sra/SRR17766310], SRR17766301 [https://www.ncbi.nlm.nih.gov/sra/SRR17766301], SRR17766300 [https://www.ncbi.nlm.nih.gov/sra/SRR17766300], SRR17766299 [https://www.ncbi.nlm.nih.gov/sra/SRR17766299], SRR17766298 [https://www.ncbi.nlm.nih.gov/sra/SRR17766298], SRR17766297 [https://www.ncbi.nlm.nih.gov/sra/SRR17766297], SRR17766296 [https://www.ncbi.nlm.nih.gov/sra/SRR17766296], SRR17766295 [https://www.ncbi.nlm.nih.gov/sra/SRR17766295], SRR17766325 [https://www.ncbi.nlm.nih.gov/sra/SRR17766325], SRR17766324 [https://www.ncbi.nlm.nih.gov/sra/SRR17766324], SRR17766323 [https://www.ncbi.nlm.nih.gov/sra/SRR17766323], SRR17766322 [https://www.ncbi.nlm.nih.gov/sra/SRR17766322], SRR17766321 [https://www.ncbi.nlm.nih.gov/sra/SRR17766321], SRR17766320 [https://www.ncbi.nlm.nih.gov/sra/SRR17766320], SRR17766319 [https://www.ncbi.nlm.nih.gov/sra/SRR17766319], SRR17766318 [https://www.ncbi.nlm.nih.gov/sra/SRR17766318], SRR17766309 [https://www.ncbi.nlm.nih.gov/sra/SRR17766309], SRR17766308 [https://www.ncbi.nlm.nih.gov/sra/SRR17766308], SRR17766306 [https://www.ncbi.nlm.nih.gov/sra/SRR17766306], SRR17766305 [https://www.ncbi.nlm.nih.gov/sra/SRR17766305], SRR17766304 [https://www.ncbi.nlm.nih.gov/sra/SRR17766304], SRR17766303 [https://www.ncbi.nlm.nih.gov/sra/SRR17766303], SRR17766302 [https://www.ncbi.nlm.nih.gov/sra/SRR17766302], SRR17766286 [https://www.ncbi.nlm.nih.gov/sra/SRR17766286], SRR17766285 [https://www.ncbi.nlm.nih.gov/sra/SRR17766285], SRR17766284 [https://www.ncbi.nlm.nih.gov/sra/SRR17766284], SRR17766283 [https://www.ncbi.nlm.nih.gov/sra/SRR17766283], SRR17766282 [https://www.ncbi.nlm.nih.gov/sra/SRR17766282], SRR17766280 [https://www.ncbi.nlm.nih.gov/sra/SRR17766280], SRR17766279 [https://www.ncbi.nlm.nih.gov/sra/SRR17766279], SRR17766293 [https://www.ncbi.nlm.nih.gov/sra/SRR17766293], and SRR17766292 [https://www.ncbi.nlm.nih.gov/sra/SRR17766292], SRR17766291 [https://www.ncbi.nlm.nih.gov/sra/SRR17766291]; for ATAC-Seq samples, SRR17766333 [https://www.ncbi.nlm.nih.gov/sra/SRR17766333], SRR17766328 [https://www.ncbi.nlm.nih.gov/sra/SRR17766328], SRR17766327 [https://www.ncbi.nlm.nih.gov/sra/SRR17766327], SRR17766332 [https://www.ncbi.nlm.nih.gov/sra/SRR17766332], SRR17766313 [https://www.ncbi.nlm.nih.gov/sra/SRR17766313], SRR17766294 [https://www.ncbi.nlm.nih.gov/sra/SRR17766294], SRR17766289 [https://www.ncbi.nlm.nih.gov/sra/SRR17766289], SRR17766288 [https://www.ncbi.nlm.nih.gov/sra/SRR17766288], SRR17766287 [https://www.ncbi.nlm.nih.gov/sra/SRR17766287], SRR17766307 [https://www.ncbi.nlm.nih.gov/sra/SRR17766307], SRR17766281 [https://www.ncbi.nlm.nih.gov/

sra/SRR17766281], SRR17766290 [https://www.ncbi.nlm.nih.gov/sra/SRR17766290], SRR17766331 [https://www.ncbi.nlm.nih.gov/sra/SRR17766331], SRR17766330 [https://www.ncbi.nlm.nih.gov/sra/SRR17766330], and SRR17766329 [https://www.ncbi.nlm.nih.gov/sra/SRR17766329]; finally, for ChIP-Seq samples, SRR17766326 [https://www.ncbi.nlm.nih.gov/sra/SRR17766326], SRR17766317 [https://www.ncbi.nlm.nih.gov/sra/SRR17766317], SRR17766315 [https://www.ncbi.nlm.nih.gov/sra/SRR17766315], and SRR17766316 [https://www.ncbi.nlm.nih.gov/sra/SRR17766316].

Other data supporting this study's findings are available within the article and its Supplementary files. Source data are provided with this paper.

Reference genome sequences (Schmidtea mediterranea S2F2 genome SmesG.v4), gene annotations (SMESG-repeat v2) and GOs for the functional annotation of the genes were retrieved from PlanMine (https://planmine.mpibpc.mpg.de/). FoxG binding site presence: USCS browser. Weight matrices for slp1 from Drosophila melanogaster (MA0458.1) and FOXG from Homo sapiens (MA0613.1) were obtained from the JASPAR CORE 2022 database [Castro-Mondragon, J. A. et al. JASPAR 2022: The 9th release of the open-access database of transcription factor binding profiles. Nucleic Acids Res. 50, D165–D173 (2022)].

As stated in the previous section, a PDF describing all the Computational Supplementary Methods (https://compgen.bio.ub.edu/datasets/2022_NatComm/2022_NatComm_BImethods.pdf) is linked from GitHub project repository together with the scripts used for the Bioinformatic analyses (https://github.com/CompGenLabUB/2022_NatComm_BImethods [DOI:10.5281/zenodo.7455255]); on the same repository a folder data contains the relevant intermediate and processed datasets.

Human research participants

Policy information about studies involving human research participants and Sex and Gender in Research.

Reporting on sex and gender	N/A
Population characteristics	N/A
Recruitment	N/A
Ethics oversight	N/A

Note that full information on the approval of the study protocol must also be provided in the manuscript.

Field-specific reporting

Please select the one below that is the best fit for your research. If you are not sure, read the appropriate sections before making your selection.

☒ Life sciences ☐ Behavioural & social sciences ☐ Ecological, evolutionary & environmental sciences

For a reference copy of the document with all sections, see nature.com/documents/nr-reporting-summary-flat.pdf

Life sciences study design

All studies must disclose on these points even when the disclosure is negative.

Sample size	No statistical analysis was used to predetermine sample size. For the ATAC-seq, ChIP-seq and RNA-seq, the number of biological samples was indicated as well as the number of animals or tissue fractions containing each biological replicate. Those were chosen based on previously reported experiments in the field. Examples: Time course RNA-seq (Wurtzel et al., Dev. Cell, 2015); qPCR (Almuedo-Castillo et al., PLoS Genetics, 2014), ATAC-seq and ChIP-seq (Neiro et al. eLife, 2022), IHC (Scimone et al. Nature, 2017), WISH (Tewari et al., PLoS Genetics, 2019), RNAi experiments (Scimone et al., Current Biology, 2018). For all experiments, sample sizes are listed explicitly in Figure Legends and/or Source Data.
Data exclusions	The RNA-seq experiment was designed to collect samples at 0, 12, 24, 48, 36 and 72 hours of regeneration (hR) in control and wnt1 (RNAi) conditions. RNAi animals were treated using a mild inhibition strategy, aiming to obtain animals with a tailless (mild) phenotype, and not bi-headed (strong). Evaluating the wnt1 expression in all the samples, we observed that one replicate of wnt1 (RNAi) at 12hR and 36hR present the same wnt1 expression levels as the controls, therefore we decided to exclude them.
Replication	For all the qualitative experiments, results are representative of at least two independent experiments, that posteriorly have been added, are indicated in Figures. Experiments in Figure 4e and Figure S8c were performed once due to time constraints and technical difficulties. RNA-seq, ATAC-seq and ChIP-seq experiments were performed once due to resource constraints, but 2-3 replicates per conditions were similar among each other (details in Computational Section Methods). qRT-PCR experiment was performed once, but similar results were observed in Figure 5b.
Randomization	For all the experiments, animals were randomly allocated into experimental groups and/or biological replicates.
Blinding	Investigators were not blinded during group allocation or analysis. This present study is mostly qualitative, describing phenotypes of different genetic backgrounds. However, qualitative data in Figures 4b, 4c, 4d, 5a, 5b, 5c, S8a, S8b and S8c was evaluated independently by at least two researchers. For automated RNA-seq, ATAC-seq, ChIP-seq and motif finding, identical instrument settings and software parameters were used for quantification and analysis of all biological replicates and genetic backgrounds.

Reporting for specific materials, systems and methods

We require information from authors about some types of materials, experimental systems and methods used in many studies. Here, indicate whether each material, system or method listed is relevant to your study. If you are not sure if a list item applies to your research, read the appropriate section before selecting a response.

Materials & experimental systems

n/a	Involved in the study
☐	☒ Antibodies
☒	☐ Eukaryotic cell lines
☒	☐ Palaeontology and archaeology
☐	☒ Animals and other organisms
☒	☐ Clinical data
☒	☐ Dual use research of concern

Methods

n/a	Involved in the study
☐	☒ ChIP-seq
☒	☐ Flow cytometry
☒	☐ MRI-based neuroimaging

Antibodies

Antibodies used	• mouse anti-synapsin (1:50, anti-SYNORF1/3C11, Developmental Studies Hybridoma Bank).<br>• anti-Smed-β-catenin2 (1:1000, Invitrogen, AC EU82825).<br>• H3K27ac polyclonal antibody (1:100, abcam, ab4729).
Validation	• mouse anti-synapsin (anti-SYNORF1/3C11, Developmental Studies Hybridoma Bank)- validation: Cebrià F. Organization of the nervous system in the model planarian Schmidtea mediterranea: an immunocytochemical study. Neurosci Res. 2008;61(4):375–84.<br>• anti-Smed-β-catenin2 (Chai et al. 2010)- Validation: Chai, G. et al. Complete functional segregation of planarian beta-catenin-1 and -2 in mediating Wnt signaling and cell adhesion. J. Biol. Chem. 285, 24120–30 (2010).<br>• H3K27ac polyclonal antibody (abcam, ab4729). Validation: the present manuscript and Jakke Neiro, Divya Sridhar, Anish Dattani, Aziz Aboobaker (2022) Identification of putative enhancer-like elements predicts regulatory networks active in planarian adult stem cells eLife 11:e79675.

Animals and other research organisms

Policy information about studies involving animals; ARRIVE guidelines recommended for reporting animal research, and Sex and Gender in Research

Laboratory animals	Schmidtea mediterranea clonal strain BCN-10. Asexual animals were cultured in glass containers and Petri dishes for experiments in planarian artificial medium (PAM) water at 20 ºC in the dark. We use an asexual strain of Schmidtea mediterranea, and therefore they are ageless.
Wild animals	No wild animals were used in this study.
Reporting on sex	The asexual strain of Schmidtea mediterranea do not develop sexual organs, therefore sex was not a parameter to considered in this study.
Field-collected samples	No field-collected organisms were used in this study.
Ethics oversight	No ethical aproval required for planarians.

Note that full information on the approval of the study protocol must also be provided in the manuscript.

ChIP-seq

Data deposition

☒ Confirm that both raw and final processed data have been deposited in a public database such as GEO.

☒ Confirm that you have deposited or provided access to graph files (e.g. BED files) for the called peaks.

Data access links<br>*May remain private before publication.*	The raw sequencing data sets generated for this research have been deposited with links to BioProject accession number PRJNA800775 in the NCBI BioProject database (https://www.ncbi.nlm.nih.gov/bioproject/). BED files for the called peaks are available from aforementioned GitHub repository (https://github.com/CompGenLabUB/2022_NatComm_BImethods), under the folder data.
Files in database submission	• anterior_atac_chip.bed.gz<br>• posterior_atac_chip.bed.gz

- ENHANCERS.bed.gz
- PROMOTERS.bed.gz

Genome browser session (e.g. UCSC)

See tracks for the "ATAC-Seq" and "ChIP-Seq" categories from https://compgen.bio.ub.edu/jbrowse/

Methodology

Replicates

Two technical replicates were used in anterior and posterior blastemas. A total of 1000 anterior and posterior blastemas were used per replicate. Groups of 100 blastemas were done at one time.

Sequencing depth

ChIP-Seq samples were sequenced HiSeq 4000 (Illumina) with a read length of 2 × 50 bp. See Table CSM.T9 (https://compgen.bio.ub.edu/datasets/2022_NatComm/2022_NatComm_BImethods.pdf) for complete stats on those samples. Raw reads were cleaned and trimmed with BBDuk.

Antibodies

H3K27ac polyclonal antibody (abcam, ab4729).

Peak calling parameters

The peak calling procedure was adapted from the ENCODE ATAC-Seq Data Analysis Pipeline but omiting the Tn5 coords shift; MACS2 was used to complete peak calling after aligning reads with bowtie and processing those alignments with samtools. Parameters are described in section 5.3 of the CSM (https://compgen.bio.ub.edu/datasets/2022_NatComm/2022_NatComm_BImethods.pdf).

Data quality

Quality checks were performed using ATACseqQC and IDR R packages, but also with deepTools (see sections 5.2.3, 5.3.1, and 5.3.2 respectively on the CSM). Plots on all those ChIP-Seq metrics as well as summary tables for IDR, computed on the aforementioned sections are available on section 9.4 of the CSM document.

Software

All software tools used for the analyses were open source, see versions listed below (and section 9.6.3 of the Computational Supplementary Methods, https://compgen.bio.ub.edu/datasets/2022_NatComm/2022_NatComm_BImethods.pdf), or custom code (released under GNU General Public License, GNU-GPL v3), which are available through the project GitHub repository: https://github.com/CompGenLabUB/2022_NatComm_BImethods
Software versions for the ChIP-Seq Bioinformatic analyses are listed here:
- MACS2 : 2.1.2
- deepTools : 3.5.1
- bowtie : 1.3.1; samtools : 1.13 (with htslib 1.13+ds); bamtools : 2.5.1
- BBDuk : 35.14
- R version 3.5.2 [DiffBind : 2.10.0]; R version 4.1.2 [ATACseqQC : 1.18.0; ChIPpeakAnno : 3.28.1; idr : 1.2]
